# Human Dead Reckoning Using a Particle Filter and Map Constraints

**DOI:** 10.3390/s26113500

**Published:** 2026-06-02

**Authors:** Joseph Russell, Jeroen H. M. Bergmann

**Affiliations:** 1 Biomedical Engineering Centre, Department of Technology and Innovation, University of Southern Denmark, 5230 Odense, Denmark; jru@iti.sdu.dk; 2 Institute of Biomedical Engineering, Department of Engineering Science, University of Oxford, Oxford OX1 3PJ, UK

**Keywords:** inertial measurement units, human tracking, dead reckoning, map constraints, particle filter

## Abstract

**Highlights:**

**What are the main findings?**

- A particle filter applied to inertial measurement data can predict human position, provided map constraints are known, and the dimensions of the map used greatly affect the distance error of position prediction.
- A map that more tightly follows the motion path does not always yield better results.

**What are the implications of the main findings?**

- It matters what kind of map is used when localisation is supported by particle filters, and care should be taken to select the appropriate size of a map.
- Using the exact boundaries of the known movement space for the map does not provide optimal performance—instead, the map of this space should be inflated by a short distance to obtain the best performance.

**Abstract:**

This paper presents an approach for tracking a person’s position by integrating inertial measurement unit (IMU) sensor values, utilising a particle filter with known physical constraints, such as a map of the space. While such approaches are well established, the effect of constraint choice in the absence of observation measurements remains poorly understood. The effect of varying the tolerance of these constraints is investigated with data collected from a Movella DOT held by a human participant walking around a 100 m running track. In particular, the dimensions of the map are varied, along with the shape. Results showed (a) that it is viable to correct particle filter error without external sensor feedback, provided constraints are provided, with a mean error of 1.8 m, and (b) there is a minimum acceptable tolerance of map width around the edge of the true activity zone, in this case approximately 8 m, and that tightening the map boundary further than this can counterintuitively lead to reduced accuracy.

## 1. Introduction

Knowing a person’s precise location is a useful tool for a range of applications. In fitness and sport science, it can be helpful for quantifying activity levels and coordination between players [1]. It could also be a valuable input for human–machine interfaces (HMIs), enabling more precise and versatile control of smart homes [2] or better inform the control of assistive medical devices [3]. In general, knowing where a person is makes it easier to predict what they are doing and what they are likely to do next. However, this is a non-trivial task that can currently only be performed with several limitations.

Inertial measurement units (IMUs) are commonly used for motion analysis in smartphones and wearable devices such as smart watches [4,5]. They provide three sensing modalities: acceleration, angular velocity, and orientation relative to a local magnetic field. These values can be combined using a Kalman Filter approach to predict global orientation, but to determine velocity and position, the acceleration must be integrated. This introduces errors that rapidly increase, making the technique unviable for longer-term use without additional information.

When localisation of this nature is performed in robotics [6], the standard approach is to use depth sensing (e.g., lidar) or computer vision as a sensing input, which is then compared to a map, which may be built in real time (Simultaneous Localisation and Mapping) [7]. This is typically used to correct the integrated position in a Kalman Filter [8].

If trying to determine a human location from a wearable device in an unmonitored space, this approach is not possible, as visual or depth sensing inputs are not available. Instead, a dead reckoning approach must be used [9]. Typically, these methods rely on specific movement states, e.g., that the person must be walking, so that Zero Velocity Updates can be used at each step [10], with recent reviews continuing to distinguish between shoe-mounted inertial navigation and unconstrained pedestrian dead reckoning [11]. In the absence of Zero Velocity Updates (as in this study, where the sensor is not mounted on the foot), more complex methods are needed to estimate step length, but again, this often relies on walking motion patterns only [12] or involves the use of additional sensors such as surface electromyography [13].

This paper therefore investigates the use of a generalised particle filter [14] method for typical, self-powered, unconstrained human motion that does not require additional sensing inputs or specific sensor placement. Instead, it relies only on IMU measurements and a set of known spatial and kinematic constraints to limit possible positions a person could be in and discard those that violate the constraints, resulting in a more accurate estimation of a person’s position.

The objective of this system is to estimate the time-varying position of an IMU sensor carried by a human participant in their right hand. This is performed in a Cartesian coordinate frame fixed to the environment, with horizontal axes aligned to the geometry of the running track and the vertical axis aligned with gravity. The particle filter estimates the sensor’s position, velocity, and accelerometer bias over time, with the output being the horizontal (2D) position of the sensor relative to the known map.

Particle filters with map-based constraints are well established [15], and recent map-aided pedestrian dead reckoning frameworks continue to use pre-built floor maps to constrain particle motion and reduce drift [16]. However, these are typically applied alongside an external measurement update (e.g., WLAN or BLE positioning). In these systems, map constraints are generally treated as a straightforward means of enforcing physical feasibility, and their impact on the particle distribution is not extensively analysed. Furthermore, the behaviour of constraints in the absence of external observation measurements remains poorly understood. In particular, this study examines how varying the size and shape of the map boundary affects localisation accuracy and demonstrates that rigid map constraints can lead to systematic bias, such that inflating the map can improve performance.

The main contributions of this paper are as follows:

(1) The formulation and testing of generalised IMU-only dead reckoning as a constraint-only particle filtering problem without external observations, relying solely on a map and knowledge of human physiology.
(2) An evaluation of how map constraint design influences localisation accuracy.

### 1.1. Sources of Error in IMU Dead Reckoning

To motivate the formulation of the Particle Filter used in this study, the primary sources of error in such integrated IMU position-based systems must first be outlined. The system must account for each of these to ensure that at least some of its particles appear close to the correct position at each time step.

#### 1.1.1. Initial Position

As the accelerometer provides only the acceleration of the sensor, the initial position appears as the unknown constant in the second integration. The particle filter must be seeded with a distribution of possible initial positions (though this could be uniform across the known map if the initial position is entirely unknown). It is intuitive that the more tightly this is constrained, the better the performance of the particle filter will be. In this study, the sensor is held by a participant, who begins at rest at a marked point on the map, so the initial position is known to a small degree of error.

#### 1.1.2. Initial Velocity

Initial velocity appears as the unknown constant in the first integration from acceleration and must again be seeded with an appropriate, context-dependent distribution. If the sensor is being used to track human motion, it can be conservatively assumed that this will be no faster than the maximum recorded sprinting speed of 12.32 m/s [17]. If the sensor is placed on the participant, and the participant is stationary when recording starts, this can be far more tightly constrained (but cannot be assumed to be zero, due to small motions in, e.g., balancing or breathing, which when integrated result in an error that grows linearly with time). As such, in this study, where the sensor is held in the participant’s hand, a small starting velocity is chosen from a uniform distribution of ±0.1 m/s for each particle.

#### 1.1.3. Accelerometer Noise

Like most sensors, IMUs are susceptible to random noise. To model this, independent noise must be added to the accelerometer readings for each particle. This can usually be assumed to be Gaussian, with mean and standard deviation given in the sensor documentation.

#### 1.1.4. Orientation Error

Accelerometer measurements are given in the local reference frame of the sensor (i.e., the IMU body frame aligned with its sensing axes) and must be rotated into the global reference frame in order to track position. IMUs are often able to estimate orientation with an internal Kalman filter, correcting integrated gyroscope readings using a magnetometer and knowledge of gravity. However, this estimate is noisy—Gaussian parameters for this are again provided in the sensor documentation. Additionally, experimentation shows there is an element of “drift error”, where changes in orientation result in an offset, reducing to zero over time. Both these factors are typically relatively small, but can produce large errors in acceleration due to the projection of components of gravity into the horizontal plane.

Multiple possible methodologies exist for dealing with this drift error, and the accuracy of the model used can have a large impact on the accuracy of the particle filter. However, such a model is specific to the sensor used, and this study aims to develop generalised methods that can be used for a range of IMUs, irrespective of weight, size, cost and measurement accuracy, provided their noise and bias characteristics are approximately known (though a less accurate sensor will result in reduced localisation performance). Developing a specific model is therefore considered beyond the scope of this study, and instead, the drift error is included in a much larger Gaussian orientation error than is provided in the specification. This naturally leads to a very wide particle spread, highlighting the need for continuous correction to prevent the particle cloud from devolving into a uniform distribution across the map.

#### 1.1.5. Accelerometer Bias

All accelerometers have a “switch-on” bias, which can vary between sessions [18]. This may be modelled as a broadly constant offset error in the recorded acceleration, though it will change slowly over time during use, according to a random walk. This is known as bias instability, and specifications for this are again normally provided in the technical documentation of a sensor. Furthermore, accelerometer bias can also change due to factors such as temperature and flexing of the sensor casing, suggesting the modelled random walk should have a larger variance than specified.

In this study, initial bias is solved for by a period of the sensor being kept at rest at the beginning of the study—the solution being the difference between the sensor measurements and the gravity vector (rotated into the sensor reference frame). The bias instability causes the particles to diverge from one another over time, and so again, constant correction is needed.

### 1.2. Error Correction

These error factors compound to produce rapidly growing errors in the estimated position of the sensor, which must be corrected for using some known information. Ideally, this would be achieved through the use of another independent sensor, such as a camera, to weigh the particles by likelihood of being correct, depending on how closely the sensor output matches the expected sensor output given the estimated position.

For the scenario of raw IMU integration, in the absence of other sensors, as could be expected in a low-cost wearable human-monitoring application, this is not possible. The only information available might be a map of the space and constraints on what is physically possible for the human.

At each time step, the particle filter can therefore simulate possible corrected sensor values given these errors, causing the particle cloud to spread, and then discard them based on those considered to be impossible. The following factors can be used:

#### 1.2.1. Map of the Space

**Figure 1:**
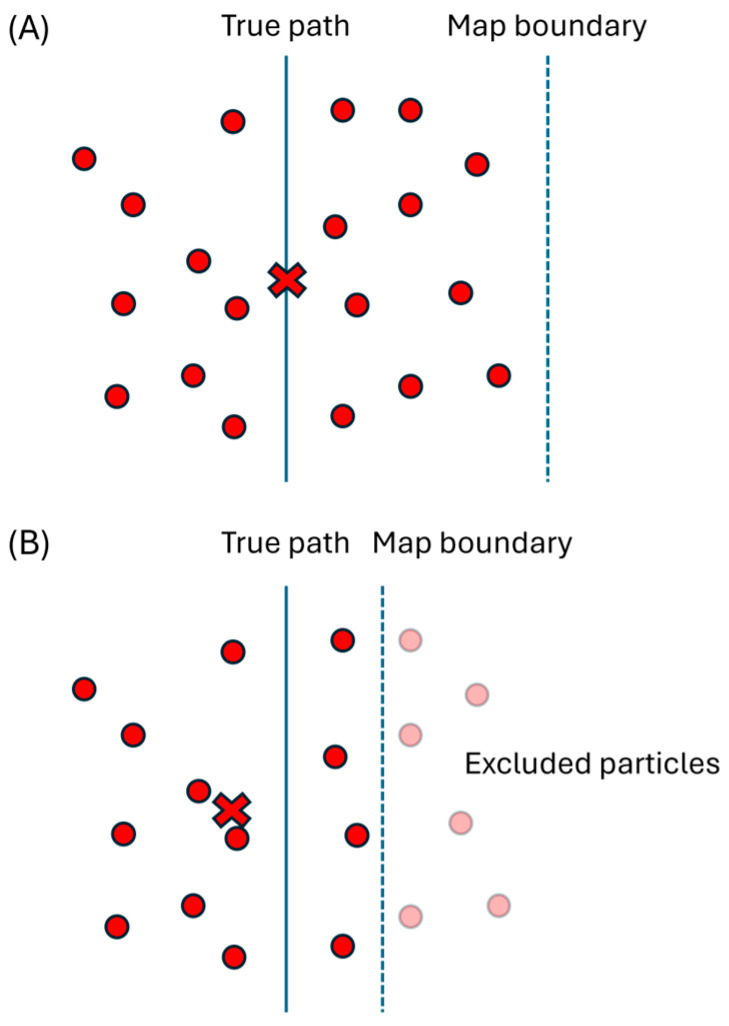
Illustration of the effect of a map boundary too close to the true path taken by the sensor. Particles are shown as red circles, with the estimated position (the mean of particle positions) shown as a red cross. In (**A**), the particle cloud is approximately symmetrically distributed around the true path, and so the estimated position lies correctly on the true path. In (**B**), the map boundary used is too close to the true path, and as such, some of the particles to the right of the true path are discarded. This causes the surviving particle cloud to be skewed to the left and the estimated position to move to the left of the true path.

Given a known map of the space the participant is moving in, particles that would place the participant outside the space can be discarded. However, this is fundamentally probabilistic. Given that much of the error is largely Gaussian, and so symmetrical around the “true” value, this means the particle cloud is always skewed away from the edge of the map—even if the true path is close to the edge—simply by the greater probability of resampling particles on the inside of the true path than on the outside, as there are more of them. As the particle cloud approaches the edge, this produces an offset error in the estimated sensor position, as shown in Figure 1.

More critically, this means the current particle cloud is also centred away from the true location, and so if the true path then moves towards the opposite edge (as it does in the experiment described in this study), the resulting integrated acceleration pushes many of the particles across the boundary. This can lead to mass particle depletion, requiring an artificial correction, relaxation of constraints, or re-initialisation of the system. This can seriously compromise particle filter performance.

This problem can be mitigated by inflating the map, deliberately allowing some particles to overshoot in order to allow the centre of the cloud to align with the true path, rather than skewing away from it. This methodology is the central investigation of this study, which will inflate the ground truth path by varying amounts to produce maps of different sizes, measuring the effect.

Even though the objective in this study is to track the participant’s position in 2D space as viewed from above, the *Z*-axis position (height above the ground) is also monitored and constrained. This is useful as a check on the orientation error: any error in pitch and roll will result in the gravity vector becoming misaligned, and so the particle will drift up or down. As such, if a particle leaves its vertical constraints, it can be assumed that the particle has suffered from large amounts of orientation error, and so its position and velocities are likely incorrect. This study assumes that the particle cannot pass more than 0.1 m below the ground and cannot rise above the height of the participant (in the experiment described in this study, it is set to 1.8 m).

#### 1.2.2. Velocity Constraints

The velocity of the sensor can also be constrained within known limits. If attached to the centre of the participant’s chest, it may be conservatively assumed that the maximum velocity of the participant is 12.32 m/s (the highest recorded speed of the current Olympic world record holder, Usain Bolt [17]). However, if positioned anywhere else, the dynamics of the human body must be considered. For instance, a human hand can have an instantaneous velocity of 20–30 m/s during a rapid motion such as throwing [19]. The maximum velocity should thus be chosen based on these factors.

For this study, the sensor was carried in the participant’s hand. The participant was instructed not to move their hand relative to the chest during the data collection. Essentially, the hand, arm and chest could be considered as a somewhat rigid body in this case. No rapid movements were recorded by the IMU during the experiment. Therefore, the maximum velocity was limited to 12.32 m/s, with particles that exceeded this discarded.

Furthermore, if the orientation of the sensor relative to the body is known, then directional velocity constraints can also be applied. For instance, humans can move forward much more quickly than they can move backwards [20], and somewhat more quickly than they can side-step [21]. Therefore, lower limits can be applied to the components of velocity in these directions.

In this study, the X axis of the sensor was always kept approximately aligned with the direction of motion, with the Z axis pointing down. As such, components of velocity in the Y axis were perpendicular to the direction of motion, and so the maximum Y axis velocity was slightly reduced to 10 m/s (a conservative maximum to reflect that side-stepping velocities are typically lower than forward sprinting, while remaining sufficiently large to maintain the filter’s generalisability beyond walking). A stricter minimum X axis velocity of −1 m/s was also set, i.e., the participant was assumed to never travel backwards faster than 1 m/s.

It may be useful to think of these velocity constraints as a “map”, just as was used for position, only now in terms of velocity. As such, it will again be expected to suffer from skew away from the edges, resulting in additional error if the true velocity is close to one of the constraints. The constraints should therefore be selected conservatively.

Based on the considerations in this and Section 1.1, the particle filter formulation used in this study is described in Section 2.

## 2. Materials and Methods

### 2.1. Data Collection

Data was collected in this study using a v2 Movella DOT (Xsens Technologies, Enschede, The Netherlands) recording at 60 Hz, initially placed on the floor for calibration and then picked up and held in the participant’s right hand before the start of data collection. This is a widely used research IMU, providing acceleration, angular velocity, and magnetic field orientation. The DOT also uses its own Kalman Filter algorithm to provide orientation data (firmware version 3.0.0).

The study is not intended to investigate human motion but rather to evaluate this methodology for tracking the position of the sensor during motion. As such, the experiment consisted of a case study of a single participant who performed three trials. General ethics was obtained to perform IMU tracking of participants in outdoor space from the Medical Sciences Interdivisional Research Ethics Committee (IDREC—R92954/RE002) at the University of Oxford.

**Figure 2:**
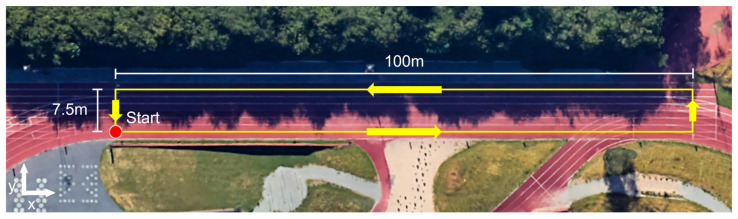
The path followed around the 100 m running track at SDU’s Odense campus. Global coordinate axes are included in the bottom left corner.

For each trial, the participant followed a rectangle outlined by the outer white lines on a 100 m section of the running track (see Figure 2), with dimensions of 100 m by 7.5 m. The sensor was initially left stationary at the bottom left corner of the rectangle, with the sensor X axis oriented along the long axis of the rectangle. After 60 s, the participant then picked up the sensor in their right hand, and stood still at the same corner for the following 60 s, the final 5 s of which are included in the recorded data. The participant then walked at a steady pace anticlockwise around the rectangle three times, ending at the starting location, facing along the long axis as they did at the beginning. The participant remained still for 5 s before the recording ended.

The initial stationary period, where the sensor was placed on the floor, was used to accurately reset the yaw heading, so that the sensor’s default X axis was aligned with the long axis of the track. A period of 60 s was allowed after the sensor was picked up before the participant began moving to allow the sensor to warm up to body temperature and reach a steady bias. Initial sensor bias for the filter was estimated from the final 5 s of this period (and subsequently allowed to drift according to bias instability).

While walking, the participant was instructed to hold the sensor in front of them with their palm facing upwards, keeping it approximately horizontal. This was performed to create optimal conditions for estimating orientation according to the sensor specification and to control operating conditions as much as possible.

Additionally, the participant had an iPhone 13 (Apple, Cupertino, CA, USA) mobile phone in their pocket, running the Gaia GPS app v2025.7 (Outside Interactive, Inc., Boulder, CO, USA) The mean absolute distance from the GPS coordinates to the true path was also computed as a reference.

### 2.2. Particle Filter

#### 2.2.1. Setup

The collected data was used as the input to a particle filter running with 100,000 particles (the maximum number used in a similar but less generalisable study on pedestrian dead reckoning with fixed map constraints [22]). This was performed offline in MATLAB R2025b (Mathworks, Natick, MA, USA), but in a causally consistent order (i.e., future measurements were never used to influence the prediction at each time step). The filter was also tested with 10,000 particles to check the difference made, but 100,000 particles was expected to be the most robust.

#### 2.2.2. Formal Filtering Problem

The objective of the filter is to estimate the time-varying state of the IMU sensor from inertial measurements and known feasibility constraints.

The global world frame was selected as a Cartesian frame fixed to the running track, with horizontal coordinates X and Y aligned with the rectangle sides and Z aligned vertical. At time step *k*, the state of particle *i* is defined as

(1) $$\begin{array}{c} x_{k}^{\left(i\right)}=\left[\begin{array}{c} p_{k}^{\left(i\right)}\\ v_{k}^{\left(i\right)}\\ \;b_{k}^{\left(i\right)} \end{array}\right]\in R^{9},\;\;\;\;\;\;\;\;\;\;p_{k}^{\left(i\right)},\;v_{k}^{\left(i\right)},\;b_{k}^{\left(i\right)}\in R^{3}\; \end{array}$$

where $p_{k}^{\left(i\right)}$ is the position, $v_{k}^{\left(i\right)}$ is the velocity and $b_{k}^{\left(i\right)}$ is the accelerometer bias.

The input at each time step is the IMU measurement:

(2) $$\begin{array}{c} u_{k}=\left\{a_{k}^{S},R_{G\to S,k}\right\}\; \end{array}$$

where $a_{k}^{S}$ is the measured acceleration in the sensor frame, and $R_{G\to S,k}$ is the orientation estimate provided by the sensor, expressed as a rotation matrix.

The process model is therefore

(3) $$x_{k}^{\left(i\right)}∽p\left(x_{k}\right|x_{k-1}^{\left(i\right)},\;u_{k})$$

where the transition consists of bias random walk, additive accelerometer and orientation noise, rotation of acceleration into the global frame, and numerical integration to velocity and position.

Unlike a standard observation-based particle filter, no external position measurement is available in this study. Therefore, there is no conventional measurement update of the form $p\left(z_{k}|x_{k}\right)$. Instead, the constraints act as the sole source of posterior correction at each time step. The map, height and velocity limits define a feasible set:

(4) $$F_{k}=\left\{i:p_{k}^{\left(i\right)}\in M(r),\;\;\;\;\;\;z_{min}\leq z_{k}^{\left(i\right)}\leq z_{max},\;\;\;\;\;\;v_{k}^{S,(i)}\in V\right\}$$

where M(r) is the horizontal map of the space, inflated by Minkowski Sum distance *r*, *z* is the particle’s vertical position, and V is the set of local velocity constraints.

This can be interpreted as a binary pseudo-measurement or constraint likelihood:

(5) $$p\left(z_{k}=feasible|x_{k}^{\left(i\right)}\right)=\{\begin{array}{c} 1,\;\;\;\;i\in F_{k}\\ \;\;0,\;\;\;\;i\notin F_{k}\;\; \end{array}$$

Particles outside the feasible set are discarded. Since no external observation is available to distinguish between feasible particles, all surviving particles are assigned equal weight:

(6) $$w_{k}^{\left(i\right)}=\{\begin{array}{ll} \frac{1}{\left|F_{k}\right|}, & i\in F_{k}\\ 0, & i\notin F_{k} \end{array}$$

Resampling is then performed from the feasible particles with uniform probability. The estimated sensor position is calculated as the mean position of the feasible particle set.

#### 2.2.3. Implementation

Initially, velocity was sampled from a uniform distribution between −0.1 m/s and +0.1 m/s in each axis (to account for body sway), and position was randomly selected from a sphere of radius 0.1 m in the bottom left corner of the path, i.e.,

(7) $$p_{0}^{\left(i\right)}∼U(S_{0})$$

where $S_{0}$ is a sphere of radius 0.1 m centred at the starting point.

(8) $$v_{0}^{\left(i\right)}∼U({\left[-0.1,\;0.1\right]}^{3})$$

Initial yaw was set with the X axis oriented along the rectangle’s long axis, plus a zero-mean Gaussian error with a standard deviation of 15 degrees, i.e.,

(9) $$\psi_{0}^{\left(i\right)}∼\;N(0,\;\sigma_{\psi }^{2}),\;\;\;\;\;\;\;\;\;\sigma_{\psi }=15{}^{\circ}$$

Initial accelerometer bias was estimated from the final 5 s of calibration by rotating acceleration due to gravity into the sensor’s local coordinates, subtracting it from the recorded accelerations, and taking the mean of the resulting values (it is assumed for this that the participant kept approximately still during this calibration), i.e.,

(10) $${\hat{b}}_{0}=\frac{1}{N_{C}}\sum_{k\in C}a^{S}-R_{G\to S}g^{G}$$

where C is the set of measurements from the final 5 s of the calibration window, and therefore $N_{C}=300$, $a^{S}$ is measured acceleration in the sensor frame, $R_{G\to S}$ is a rotation matrix from global to local (sensor) coordinates and $g^{G}$ is the gravity vector in the global frame.

Initial bias was normally distributed around the estimate taken in Equation (10), with a standard deviation of 0.1 m/s^2^, i.e.,

(11) $$b_{0}^{\left(i\right)}∼N({\hat{b}}_{0},\sigma_{b}^{2}I),\;\;\;\;\;\;\;\;\;\sigma_{b}=0.1\;m/s^{2}$$

At each time step, bias was allowed to fluctuate with a random walk (a standard representation of accelerometer bias instability [18]), with an increment in each axis each time step sampled from a normal distribution with standard deviation 0.03 mg/s^1/2^, as per the bias stability given in the sensor manual [17]:

(12) $$b_{k+1}^{\left(i\right)}=b_{k}^{\left(i\right)}+w_{b,k}^{\left(i\right)},\;\;\;\;\;\;\;\;\;w_{b,k}^{\left(i\right)}∼N(0,\sigma_{w}^{2}I)$$

(13) $$\sigma_{w}=0.03\times 10^{-3}\times g/\sqrt{f_{s}}$$

where g is acceleration due to gravity and $f_{s}$ is the sample rate—in this case, 60 Hz.

At each time step, normally distributed accelerometer noise with a standard deviation of 120 µg/$\sqrt{Hz}$ was added to each axis of acceleration data. For the X and Y axes, denoted by $\sigma_{axy}$, this was multiplied by the square root of the X and Y axis sensor bandwidth of 324 Hz. For the Z axis, denoted by $\sigma_{az}$, this was multiplied by the square root of the Z axis sensor bandwidth of 262 Hz. Accelerometer bias was subtracted from these readings.

(14) $${\tilde{a}}_{k}^{S,(i)}=a_{k}^{S}-b_{k}^{\left(i\right)}+w_{a,k}^{\left(i\right)},\;\;\;\;\;\;\;\;\;w_{a,k}^{\left(i\right)}∼N(0,\Sigma_{a})$$

(15) $$\Sigma_{a}=diag(\sigma_{axy}^{2},\sigma_{axy}^{2},\sigma_{az}^{2})$$

Sensor specifications for orientation error are given in the manual [23] as standard deviations of 2° in the heading and 1° in inclination; these were multiplied by a factor of 8 to account for orientation drift error, which is not documented in the manual. This scaling factor was selected empirically using a pilot data set as the minimum level needed to prevent widespread particle depletion. The qualitative behaviour observed in this study was not found to depend critically on the exact value of this parameter. Details on this can be seen in Appendix A.

Normally distributed noise was accordingly added to the sensor orientations.

(16) $$R_{S\to G,k}^{\left(i\right)}=R_{S\to G,k}∆R_{k}^{\left(i\right)}$$

(17) $${∆R}_{k}^{\left(i\right)}=R_{z}(\psi_{0}^{\left(i\right)})R_{x}(\delta \phi_{k}^{\left(i\right)})R_{y}(\delta \theta_{k}^{\left(i\right)})R_{z}(\delta \psi_{k}^{\left(i\right)})$$

where $\psi_{0}^{\left(i\right)}$ is the initial yaw offset for particle i, found in Equation (9), and $\delta \phi_{k}^{\left(i\right)}$, $\delta \theta_{k}^{\left(i\right)}$ and $\delta \psi_{k}^{\left(i\right)}$ are the roll, yaw and pitch perturbations of particle *i* at time step *k*.

(18) $$\delta \phi_{k}^{\left(i\right)}∼N\left(0,\;\sigma_{\phi }^{2}\right),\;\;\delta \theta_{k}^{\left(i\right)}∼N\left(0,\sigma_{\theta }^{2}\right),\;\;\delta \psi_{k}^{\left(i\right)}∼N\left(0,\sigma_{\psi }^{2}\right)$$

The accelerations were then rotated by the Sensor-to-Global rotation matrix to produce global accelerations:

(19) $$a_{k}^{G,(i)}={\tilde{R}}_{S\to G}^{\left(i\right)}{\tilde{a}}_{k}^{S,(i)}$$

These were integrated to velocity, and then to position:

(20) $$v_{k+1}^{\left(i\right)}=v_{k}^{\left(i\right)}+\frac{1}{2}(a_{k}^{G,\left(i\right)}+a_{k+1}^{G,\left(i\right)})∆t$$

(21) $$p_{k+1}^{\left(i\right)}=p_{k}^{\left(i\right)}+\frac{1}{2}(v_{k}^{\left(i\right)}+v_{k+1}^{\left(i\right)})∆t$$

At each time step, the particles’ velocities were rotated back into local coordinates:

(22) $$v_{k}^{S,(i)}={\tilde{R}}_{G\to S}^{\left(i\right)}v_{k}^{G,(i)}$$

These were checked against the velocity constraints for expected human motion (defined as 12.32 m/s forward, 10 m/s sideways and 1 m/s backwards).

The key research question of this study was to investigate the effect of varying the map. To answer this, the map of the space used to limit the particles’ positions was controlled by inflating the true path as defined by the white lines with a Minkowski Sum by distance r—a concept commonly employed in robotics planning for obstacle avoidance, albeit in the opposite direction [24]. This distance was varied from 0 to 16 m in increments of 4 m to investigate the effect of changing the map dimensions. Additionally, a circular map with radius 58 m, centred on the middle of the ground truth path, was investigated, along with an “unbounded” map—a rectangle with a Minkowski Sum distance of 1000 m.

These constraints were used to define the feasible set of particles, as seen in Equation (4).

Any particles violating these velocity and spatial constraints were discarded and resampled from the set of particles that satisfy the constraints. As there are no observations, no surviving particle was considered to be any more likely to be correct than any other, and therefore all resampling weights were uniform, as seen in Equations (5) and (6). The overall estimated position of the sensor at each time step was calculated as the mean of the positions of all feasible particles.

For each of the three recorded trials, the particle filter was run 10 times using the same IMU input data but different random seeds for particle initialisation, random error and resampling. These repeated runs were used to characterise the variability of the algorithm due to its internal randomness, rather than to represent variation across participants. The number of repeats was chosen as a practical balance between computational cost and obtaining a representative estimate of variability. As each trial consisted of three laps around the track, this resulted in 30 particle filter runs, corresponding to 90 laps in total.

### 2.3. Accuracy Metric

No method was available to obtain a reliable ground truth for the specific position of the participant during data collection. The only certain positional information is that the participant followed the lines on the running track as shown in Figure 2 and completed three anticlockwise laps of this 100 m by 7.5 m rectangle before coming to a stop. Therefore, the accuracy metric used for the particle filter is the Euclidean distance of the estimated position from the nearest edge of the rectangle at each time step, which may be more precisely referred to as path adherence. This is distinct from the Minkowski sum distance, which is an independent variable controlling how much the map is inflated. The mean across all time steps is then used to calculate the accuracy metric for comparison. While this metric is applicable in this scenario, care should be taken, as it is, of course, perfectly possible for a system to obtain a 100% path adherence simply by predicting that the participant remained still in one corner of the map.

To ensure this did not happen, a simple check was implemented: the map was divided into four equal quadrants, and a particle filter run was only accepted as valid if all four quadrants were visited at least once. This check was satisfied in all cases.

## 3. Results

**Figure 3:**
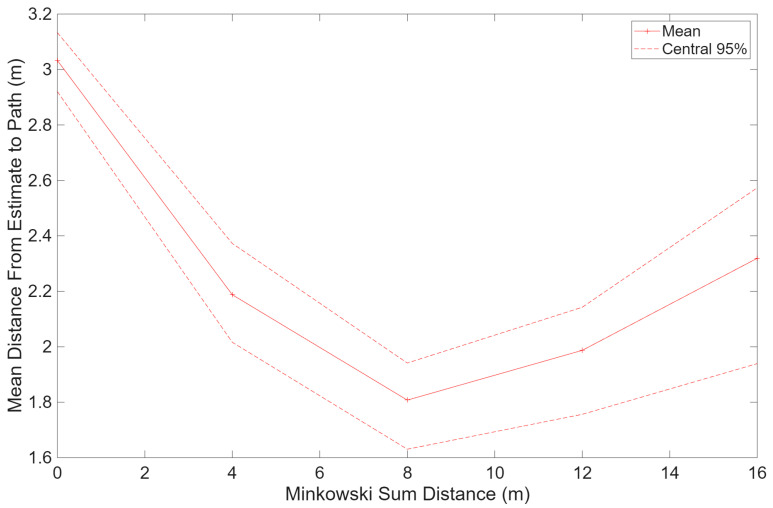
The mean distance from the ground truth rectangle across all time steps against the Minkowski Sum distance. Across all 30 repeats (10 runs of the particle filter on each trial), the mean of the per-run mean errors is shown, along with the central 95%.

The Minkowski Sum distance of the map around the path was varied from 0 m to 16 m, and the particle filter algorithm was re-run, with results shown in Figure 3. A distance of 8 m was found to have the lowest mean error at only 1.8 m, with error increasing monotonically for lower and higher distances than this. For comparison, the GPS app achieved a mean absolute distance of 3.7 m from the ground truth path—a larger error than the particle filter for all tested distances.

It should be noted that this value is specific to the experimental conditions of this study, including the size of the track and the noise profile of the sensor, and should not be interpreted as a universal optimal value.

**Figure 4:**
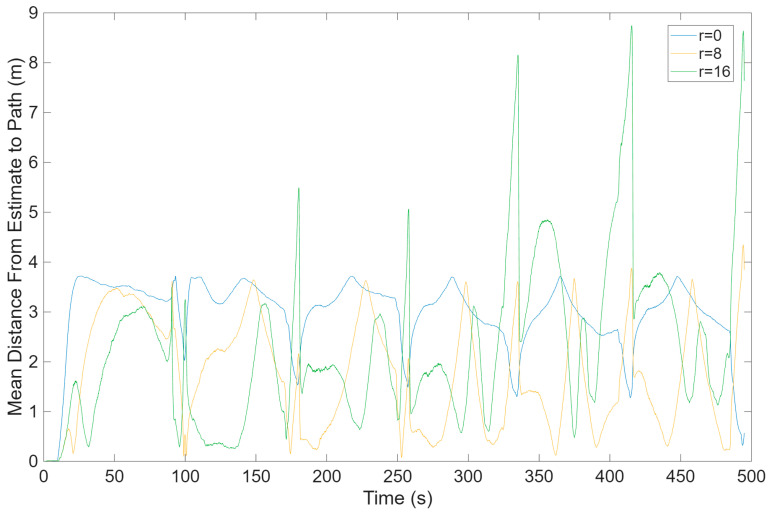
The mean distance from the ground truth rectangle at each time step, averaged across all 10 repeats of running the particle filter on a single trial, with varying values of *r*, the Minkowski Sum distance.

Shown in Figure 4 is the performance over time for Minkowski Sum distances of 0 m, 8 m and 16 m, averaged over all re-runs of the particle filter for a single trial. This is intended as an illustration of error behaviour, and is not necessarily representative of all trials. While a distance of 8 m had the lowest mean error, it should be noted that this was not consistent at all time steps, with the 0 m and 16 m distances showing lower error at different times.

**Figure 5:**
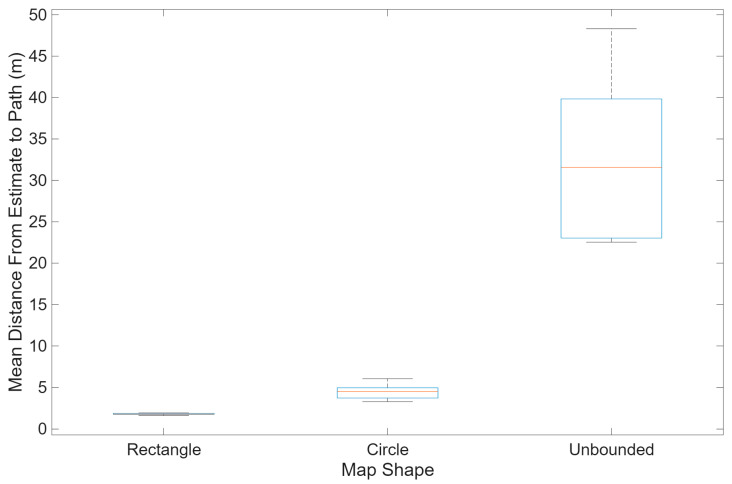
Box and whisker plots for the mean distance from the ground truth rectangle, averaged across all time steps and repeats, for three different maps: a rectangle (with Minkowski Sum distance of 8 m), a circle (with radius 58 m, centred on the centre of the path), and unbounded (with effective distance of 1000 m).

A Minkowski Sum distance of 1000 m was also tested to identify a baseline for an effectively unbounded map. This was compared to the best-performing distance for a rectangular map, and to a circular map with a radius of 58 m, in Figure 5. The rectangular map had by far the lowest error of the three. There was no overlap between the ranges of any of the three map shapes across repeats.

**Figure 6:**
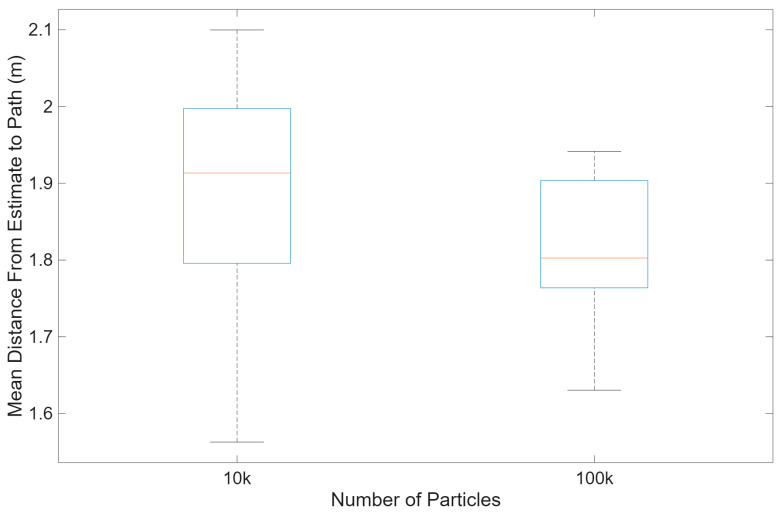
Box and whisker plots for the mean distance from the ground truth rectangle, averaged across all time steps and repeats, for 10,000 and 100,000 particles, both with a Minkowski Sum distance of 8 m.

Finally, the effect of reducing the particles by a factor of 10, to 10,000, was investigated, with results shown in Figure 6. The median error, mean error, variance and range were all lower with 100,000 particles than with 10,000.

## 4. Discussion

It might reasonably be assumed that to produce the highest possible accuracy estimation of position, all particles that violate constraints, and therefore have zero probability of being correct, must be immediately discarded. Indeed, classic observational particle filter models would assign impossible positions a zero weighting and eliminate them during resampling [8,25]. However, the results show that this is not the case, and that there is benefit in using an expanded map—particularly so if the path is expected to pass close to the map boundaries during the activity, as it did by design in this study.

It was expected that the map boundary of zero would not produce the best performance—partly because of the skewing effect described in Section 1, but also more intuitively because any particles, even the slightest distance on the outside of the true path, would be discarded, making it in practice impossible for any particle to correctly follow the true path. This was observed in the results, with the 0 m map showing the highest error across all bounded rectangles and also the lowest variance, meaning its performance was the most consistently poor.

Inflating the boundary by up to 8 m caused the error to monotonically decrease, as the skew effect was reduced. However, this is a trade-off, as decreasing the skew by increasing the Minkowski Sum distance also reduces the ability of the map to discard incorrect particles. The best balance between these factors was achieved in this case at approximately 8 m, after which the error increased with distance. The circular boundary was shown to have approximately double this error, and the effect of removing the map boundaries altogether can also be seen in Figure 5, where the overall error increased by an order of magnitude from the 8 m map.

It should be noted that this value of 8 m is specific to the experimental conditions of this study, including the scale of the track, the motion characteristics of the participant and the noise profile of the sensor. The optimal inflation distance is expected to fundamentally depend on the magnitude of uncertainty in the particle filter. In general, higher uncertainty will require a larger inflation distance, because when the true path lies close to the boundary, the particle cloud must be allowed to extend beyond the nominal map in order for its mean to remain correctly aligned with the boundary.

A practical approach is therefore to select this parameter based on the expected spread of the particle cloud, either through a separate calibration stage or by adapting it dynamically based on observed spread. The ideal inflation distance should be chosen such that, when the particle distribution is correctly centred on the true position, the boundary does not truncate the particle cloud.

In practical applications, the true path is not known beforehand. Instead, map constraints would typically be derived from environmental information such as building layouts, floor plans and sports pitches [16]. Ideally, the uninflated map would represent the full region within which the participant is able to move, and it is this region that should be inflated with the Minkowski Sum. The use of a known path in this study is a controlled means of isolating the effect of map boundary design on particle filter performance, with a boundary intentionally placed close to the path to show this effect. The further away the true path is from the boundary, the lesser the impact will be, such that boundary inflation may be less critical when the expected path lies well within the interior of the map. This is an alternative interpretation of the result in Figure 3.

At the optimal distance of 8 m observed in this study, similar path adherence was seen when the number of particles was reduced by a factor of 10, down to 10,000, albeit with a slightly higher mean and larger variance. This has implications for performance, as a particle filter with a tenth of the particles runs at least 10× faster, and requires only a tenth of the memory, making it far more viable for practical deployment on a mobile device.

In all cases for the bounded rectangular map, the particle filter outperformed the baseline provided by the GPS app, with a minimum error of only 1.8 m compared to the GPS error of 3.7 m—a more than 50% reduction—supporting this methodology as a suitable method of human dead reckoning.

### 4.1. Limitations of the Study

The lack of a specific ground truth of the participant’s position over time is a fundamental limitation of the study, and one that is very challenging to resolve on this scale in practice. A distance of 100 m is large for a standard optical tracking system to work over, and GPS cannot produce anywhere near the precision required for a useful ground truth. Nonetheless, it would be possible in future studies to combine several modalities to overcome this limitation.

Furthermore, this study used only a single participant. While this was sufficient to investigate the research problem of how accurately the system was able to follow the path, and how this is affected by the map used, it may not be generally representative of human behaviour. Although repeated runs of the particle filter on the same three recorded trials were informative about the algorithm’s stochastic variability, they do not constitute independent experimental repeats and should not be interpreted as evidence of multi-participant variability. A larger-scale study would be able to investigate this more thoroughly.

Finally, the particle filter was run with 100,000 particles, requiring sizeable computing resources and time to execute. This was performed to simplify the problem, removing the question of whether enough particles had been simulated to create a diverse enough particle cloud. However, this is not practical for general use—future tests should use fewer particles, or even an adaptive particle filter, with numbers of particles varying depending on context [26].

### 4.2. Suggestions for Future Work

By simulating the errors described above and discarding particles at each time step based on known constraints, a system has been developed to track human position using only an IMU sensor, without requiring feedback from additional sensing such as a camera. However, the accuracy of this is relatively low, and further techniques should be explored to improve it.

#### 4.2.1. Reduce Sensor Error

Error in the system primarily comes from inaccuracies in the sensors—indeed, if the measurements of the sensors were perfect, only a single particle would be needed. More accurate IMUs than the DOT are available, with lower noise profiles, and future studies should explore these. Additionally, the problem of orientation drift may arise from the DOT’s onboard Kalman Filter, and alternative algorithms could be developed and tested to reduce this further.

#### 4.2.2. Use More Sensors

This study used only one IMU to predict position. The addition of more IMUs, each connected to its own particle filter, and averaging the results, may reduce overall error. This will only be effective if the errors are independent from sensor to sensor—true if it is mostly random Gaussian noise, but untrue if systematic factors like an external magnetic field or temperature fluctuations are at fault.

## 5. Conclusions

This study has shown that (a) it is possible to generally predict human position from IMU measurements using a particle filter to a much higher degree of path adherence than GPS for “smaller” paths, so long as a map is known, and (b) that the shape and dimensions of this map have a major impact on the accuracy of this system, with a bounded rectangular map inflated by 8 m found to be the best performing of tested maps under the conditions of this study.

It is hoped that these findings will form a foundation for a re-examination of map practices in general particle filter usage, particularly in dead reckoning, so that these systems may provide a more accurate estimation of human position for the development of HMI devices in the future.

## Figures and Tables

**Figure A1:**
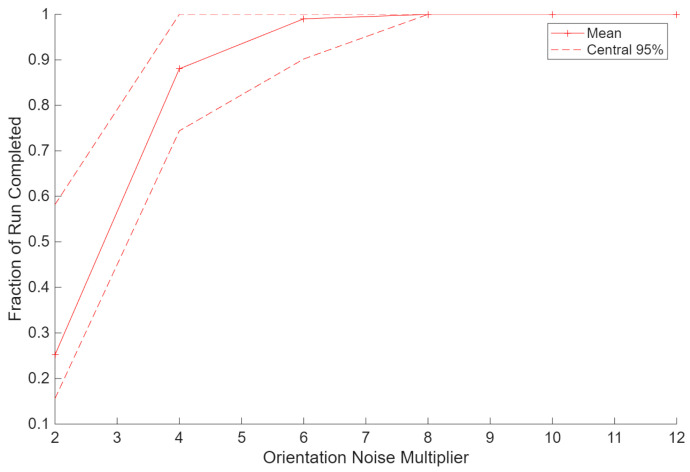
Calibration plot of the mean fraction of the run completed before particle depletion occurred vs. the orientation noise multiplier used. Tested on a withheld pilot trial with 10 particle filter repeats.

**Figure A2:**
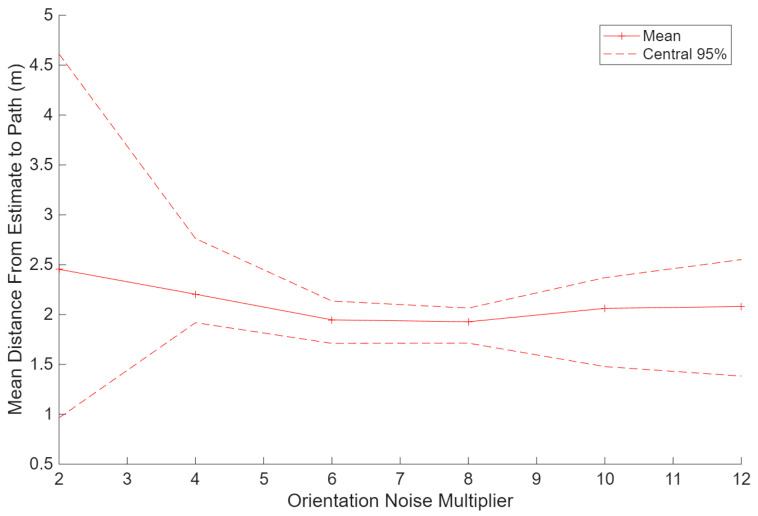
Calibration plot of the mean distance from the estimate to the path (up until particle depletion occurred) vs. the orientation noise multiplier used. Tested on a withheld pilot trial with 10 particle filter repeats.

## Data Availability

The raw data supporting the conclusions of this article will be made available by the authors upon request.

## Abbreviations

The following abbreviations are used in this manuscript:

IMU	Inertial Measurement Unit.
HMI	Human Machine Interface.

## Appendix A. Calibration Results

In Section 2.2, the orientation error provided in the manual was multiplied by a factor of 8 in order to capture undocumented orientation drift. This factor was set empirically based on a withheld repeat of the dataset as the lowest acceptable value that reliably did not result in particle depletion. The effect of varying this is shown in Figure A1 and Figure A2, which, respectively, show the fraction of the run completed before total particle depletion occurred (i.e., no valid particles remained) and the path adherence for the fraction of the run that was completed. The particle filter was re-run 10 times.

Given these, it can be observed that multiplying factors of 8 and above showed no recorded instances of particle depletion and that path adherence was broadly consistent regardless of the specific value chosen.
